# Prioritizing Patient Reported Outcome Measures (PROMs) to use in the clinical care of youth living with mental health concerns: a nominal group technique study

**DOI:** 10.1186/s41687-024-00694-z

**Published:** 2024-02-21

**Authors:** Kalpana Thapa Bajgain, Justino Mendoza, Farwa Naqvi, Fariba Aghajafari, Karen Tang, Jennifer Zwicker, Maria-Jose Santana

**Affiliations:** 1 Department of Community Health Sciences, University of Calgary, Calgary, Alberta Canada; 2 General Psychology, Capella University, Minneapolis, MN USA; 3 Department of Medicine and Community Health Science, University of Calgary, Calgary, Alberta Canada; 4 School of Public Policy, University of Calgary, Calgary, Alberta Canada; 5 Department of Pediatrics, University of Calgary, Calgary, Alberta Canada; 6 Faculty of Kinesiology, University of Calgary, Calgary, Alberta Canada; 7 Alberta Children’s Hospital, 2888 Shaganappi Trail NW, Calgary, Canada

**Keywords:** Patient-reported outcome measures, Feasibility, Relevance prioritizing, Nominal group technique, Youths, Patients/caregivers

## Abstract

**Background:**

In the past few decades, particularly in the mental health setting, there has been growing interest in using Patient Reported Outcome Measures (PROMs) to assess the efficacy of the treatments in healthcare systems. Despite recent initiatives for global harmonization, there remains a lack of consensus on which PROMs are best practice and appropriate. Engagement of the service users, such as patients and family members/caregivers, is vital at this stage to ensure the selected PROMs are feasible, relevant, and acceptable to them. This study aimed to prioritize PROMs by youth and family/caregiver based on feasibility, relevance, and overall importance to be used in the clinical care of youth living with anxiety and/or depression.

**Methods:**

Ten validated and widely used PROMs were presented to the patients and family/caregivers. Nominal group techniques were employed to prioritize the PROMs based on feasibility, relevance, and overall importance.

**Results:**

For patients and families/caregivers, the PROMs, Revised Child Anxiety and Depression Scale (RCAD 25), and The Young Person’s Core (YP-CORE) were the highest priorities. Both felt that RCAD 25 was comprehensive, short, easy, and quick to complete, whereas regarding YP-CORE, patients and family/caregivers thought it was also short and relevant. Due to some specific concerns, the Strength and Difficulties Questionnaire and Child Health Questionnaire were the lowest prioritized by patients and family/caregivers.

**Conclusion:**

It is of utmost importance that patient’s and family/caregivers’ voices or opinions are considered while selecting and implementing PROMs in mental health settings. Our study provides practical recommendations around measures best suited to achieve this.

## Background

In Canada, 1.2 million children and youth are impacted by mental illness, with about 80% of mental health problems starting before age 26. However, less than 20% will receive the necessary care [1, 2]. Mental illness can have long-term implications, including increased unemployment rates, criminal activity, high school dropout, and unstable income [3]. Based on the Canadian Health Survey in 2019, 7.4% of youth aged 12–17 encountered anxiety disorders, yet 3.8% stated mood disorders [4]. In Canada, 75% of those with mental illness never seek specialized treatment services, implying a lack of adequate treatment and a gap in youth mental health services [5]. The urgent need for adequate mental health support and services for youth has been further exacerbated by the COVID-19 pandemic [6]. With the information assessed, the economic hardship of mental health illnesses in Canada conservatively costs $51 billion yearly [7]. Even though there has been an increase in the availability of mental health services in recent eras, the service delivery systems are failing to lessen the prevalence of anxiety and depression in youth [8].

In the past few decades, particularly in the mental health setting, there has been growing interest in using Patient Reported Outcome Measures (PROMs) to assess the efficacy of the treatments in healthcare systems [9]. PROMs are standardized, validated self-reported questionnaires completed by patients to determine whether the impact of the healthcare interventions and practices enhances the patient’s health and quality of life [10]. Routine use of PROMs in clinical care may facilitate better identification of the patient’s unmet needs, enhance patient/family communication, quality management, evaluation of treatment outcomes, and improve patient outcomes [11].

Despite these benefits, there are barriers to integrating PROMs into clinical practice. These include logistical, cost, technological, workflow, and privacy considerations [12, 13]. In addition, McNeill et al. showed that most of the PROMs that have been developed and integrated into practice have been done so without direct input from the service users, resulting in questions about their relevance and appropriateness, particularly from the perspective of patients [14]. It is possible that the PROMs that researchers or clinicians choose may not measure the outcomes that are vital to the target group being studied [15].

A recent review reported inconsistencies in using PROMs in clinical care could potentially be deviations in the intended effects and introducing measures related to weakness [16, 17]. The variations and gaps in the data limit the ability to identify the best practices, guide quality improvement initiatives, and ability to compare various clinical care models [18]. Despite recent initiatives for global harmonization, there remains a lack of consensus as to which PROMs are best practice and appropriate [19]. Engagement of the service users, such as patients and family members/caregivers, is vital at this stage to ensure the selected PROMs are feasible, relevant, and acceptable to them.

This study aimed to prioritize PROMs by youth and family/caregiver based on feasibility, relevance, and overall importance to be used in the clinical care of youth living with anxiety and/or depression. Findings from this study can be widely useful for helping to streamline measurement-based care approaches in youth clinical mental health service delivery and will inform the Measurement-Based Care (MBC) program at a new community mental health center, “The Summit Center,” in Calgary, Canada [20].

## Methods

See Fig. 1.

**Fig 1.**
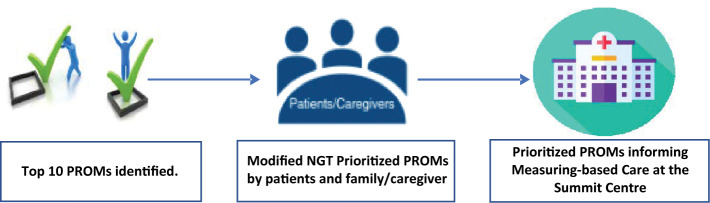
Prioritizing PROMs for youth living with anxiety/depression by patients and family/caregivers to be implemented at the Summit Centre [20]

### Study design

Modified Nominal Group Technique (NGT) [21–23] was used to prioritize PROMS. The NGT was conducted with patients living with anxiety and depression and their families/caregivers. Delbecq and Van de Ven introduced NGT as a process to identify and develop the appropriate solutions to solve strategic problems [24]. It is a stepwise consensus-building process resulting in a list of collective priorities. This approach permits empowering individuals who might otherwise be excluded from decision-making and provides equal opportunity for each group member to participate in the discussion [21]. For instance, NGT has been successfully used to include people with learning disabilities and also with people living with mental illness [22, 25].

Additional benefits of using the NGT include: (1) it is time efficient and cost-effective; (2) priorities can be elicited in a short period; (3) it requires limited budget and preparation; (4) it inspires equal input from various viewpoints [23]. During the NGT, qualitative and quantitative data were collected to present our findings more thoroughly.

### Setting

The Summit Centre is a mental health center in Calgary, Alberta that opened in March 2023. It is dedicated to assisting young people aged between 0–18 years old who are struggling with mental health problems. The services offered are (1) child and youth Mental Health walk-in services, (2) a day program for helping youth transitioning from hospital to home, (3) Intensive Community treatment services aimed to avoid the demand for hospitalization, offer short-term, intensive therapy, including individual, group and family therapy to the youth with escalating mental health concerns [20].

### Participant recruitment and selection

We recruited youth diagnosed with anxiety and/or depression and family/caregivers living in the Calgary area between January and April 2023. Interested participants were identified via the Mental Health Research for Kids (MHR 4 Kids) Research registry [20]. The MHR 4 Kids Research registry is maintained by the Summit Center to connect children, adolescents, and families who are interested in participating in mental health research with researchers to decrease recruitment barriers.

We targeted approximately five to twelve participants, in line with the standards of the NGT practice [26], where sample sizes range from 2 to 14 [23]. Inclusion criteria included youth 12–17 years of age who have been through anxiety and/or depression and family members/caregivers of patients who have been receiving mental health services. The single exclusion criterion was the inability to provide full informed consent. Separate nominal groups were conducted for family/caregivers and patients, as the youth patient participants may be hesitant to share their perspectives with adult participants.

Participants received a package that included an information sheet describing the aim and objective of the study; a consent form; a socio-demographic questionnaire that was used to describe participant characteristics; a pre-identified top 10 PROMs (see Sect. “Pre-identification of PROMS”) with ranking criteria (e.g., feasibility, relevancy, and overall importance); and a description of the NGT process. This package was sent a week prior to the NGT session.

Participants signed a consent form and completed the socio-demographic information form prior to participating in the NGT session. We conducted two separate virtual NGT sessions with patients and family members/caregivers, lasting approximately two hours each. Both were co-facilitated by two research members (KT and FNS) and one youth patient-partner (JM). Ethics approval was acquired from the University Health Research Ethics Board [REB22-0880] at the University of Calgary.

### Pre-identification of PROMS

The NGT is a phase of a more extensive multi-phased mixed-method study that includes the following steps that served to identify the PROMS to be discussed within the NGT.

#### Systematic literature review to identify PROMs

We conducted a systematic literature search to identify the PROMs that have been used in youth living with mental health concerns. MEDLINE, PubMed, and PsycINFO databases were searched for publication before 2000. Searches were limited from 2000 because the integration of PROMs in routine clinical care was initiated after 2000. The search terms include, but are not limited to “Patient-reported outcome measures”, “routine outcome assessment,” “self-reported outcome,” “patient outcome assessment,” “health-related quality of life,” Mental health,” “mental illness,” “mental disorder,” “mood disorder,” “Schizophrenia,” “eating disorder,” “psychological disorder,” “Depression” or “bipolar,” “anxiety,” and “self-harm.” Articles were selected for review if they: (1) used one or more PROMs (we included PROMs as measurement tools that are validated for use in different settings); (2) were conducted in a population <18 years of age with at least one MHC with a formal diagnosis according to the DSM 5 [24]; (3) was peer-reviewed; and (4) was published in English and the full text was available. Measures could be completed by children, parents, or both. Four team members (KTB, MMA, KW, and FN) independently screened titles and abstracts of all studies against our predetermined inclusion and exclusion criteria. The studies that did not meet the inclusion requirements were eliminated. Discrepancies were discussed and resolved by senior authors (MS and JZ) where necessary.

We identified 28 PROMs that have been used in youth living with mental health concerns. This work has been published [16].

#### Mapping evidence to the existing resources available and consultation with a family advisory group

The finding from the systematic review was mapped to the PROMs considered by the clinical team. The clinicians considered PROMs recommended by the International Consortium for Health Outcomes Measurements Set of Patient-Centered Outcome Measures for Children and Young People with Anxiety and Depression (ICHOM) [18]; PROMs identified in an Alberta pediatric measurement scan [27, 28] and a Measurement-based care (MBC) environmental scan Mental Health (measures identified in use by major Canadian child and youth mental health clinics and pediatric mental health programs across Canada), as well as a scoping review identifying PROMs in youth living with neurodevelopmental and mental health conditions [29]. After the mapping, the PROMs were reviewed by the researcher and youth partner and, through consultation, reduced to the top 10. These top 10 evidence-based and clinician-informed PROMs were then presented to the youth and family advisory council at the Summit Center [20] for consultation. During the consultation, the advisory group was asked about the appropriateness and general thought on the use of these PROMs in their clinical care (i.e., acceptable, feasible, relevant to the patient’s need). These findings are in a manuscript under submission.

This manuscript describes the project’s final phase, prioritizing the top 10 PROMs by patients and family/caregivers to inform the MBC program at the Summit Centre.

### Modified NGT

The objective of the modified NGT was to prioritize the pre-identified PROMs based on relevance, feasibility, and overall importance. We started the NGT session by introducing the team members and participants. The facilitator then described the purpose of the study, the NGT process, and a list of the top 10 PROMs with a description of each PROMs for prioritization. The sessions were audio-recorded and transcribed verbatim. We conducted a content analysis of the transcript.

**Fig. 2:**
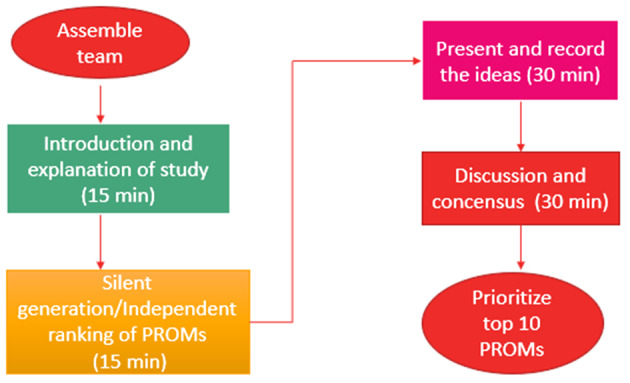
Study design: modified nominal group technique process

Typically, NGT comprises four stages: (1) silent generation of ideas, (2) Round robin, (3) Clarification and discussion, and (4) voting/ranking [20, 23]. The NGT process is a flexible consensus method that can be altered to fit the participant’s voice, the study’s purpose and goal, and the aim of generalizability [23, 26, 30]. In this study, the silent generation round was replaced with the explanation stage, where participants voted on the pre-identified PROMs based on relevance to the patient (domain coverage, e.g., pain, mental health or wellbeing, physical, social functioning, etc.), feasibility, (e.g., number of questions, timely completion, mode of administrations) and overall importance (Fig. 2). The list of PROMs for prioritization was displayed in Microsoft Forms for all participants to view in real-time. Participants were then asked to complete the survey, prioritizing PROMs from 1 to 10, with one being the least important and 10 being the most important. Prioritization was done anonymously using Microsoft Forms. Each participant received a unique sheet with the top 10 PROMs in one column and prioritizing values (1–10) listed in the adjacent column. Participants were then asked to rank each PROM based on the criteria provided, assigning only one number per PROM without sharing this with others. This step was crucial because it allowed participants to rank the PROMs independently before considering other perspectives that were revealed in the group discussion. Once the individual ranking was completed, the summary sheet that included the list of PROMs was reorganized based on the order of priority at the group level for further discussion.

The next step after the voting round is discussing and sharing ideas. Here, there was a group review of the aggregate score of the initial rankings. These aggregate scores were displayed on a summary sheet and presented to participants. The participants were asked to explain their priorities and any comments related to the summary sheet. After this first round of discussion, participants had an opportunity to reconsider their initial ranking of the PROMs based on the discussion. Participants were not pressured to change their ranking or reach a consensus. The facilitators made sure that each participant had a chance to contribute to the discussion.

Throughout the NGT session, the youth researcher, with previous experience engaging in mental health research, was actively involved in making sure the research activities and outputs matched the youth patient’s perceptions and feedback and in creating a supported and comfortable environment for the youth patient to share their views and experiences as youth researcher share the similar experiences and language [31]. This role was crucial to ensure patients’ voices were central to the discussions and reduce the power imbalances during the NGT process.

### Data analysis

To boost our interpretation of the findings, we used a mixed methods approach to analyze the result [23]. Participants’ descriptive characteristics were summarised using numbers and percentages. To determine the priority of each PROM, we calculated the median ranking and interquartile range for each prioritized PROM, as well as the minimum and maximum range ranking. Analysis was conducted using Microsoft Excel. We also created a cluster bar chart to compare the ranking of PROMs across patients and family/caregivers. A verbatim transcript was produced from the Zoom meeting recording and analyzed thematically.

### Results

There were thirteen participants (five patients and eight family/caregivers). Five patients living with anxiety/and or depression participated in the NGT session, as seen in Table 1. Most of the youth participants were female (60%), and 40% of them were non-binary; 80% were older than 15 years, and only one participant was aged between 12 and 14 (20%). Eight families/caregivers aged between 40 and 49 participated in the NGT session; seven (87.5%) were female.

**Table 1:** Characteristics of participants

Family/caregiver (N = 8) % (n)		Patients (N = 5) % (n)		
**Gender**				
Female	87.5% (7)		Female	60% (3)
Male	12.5% (1)		Non-binary	40% (2)
**Age, yr.**				
40–49	75.0% (6)		12–14	20.0% (1)
50–51	25.0% (2)		15–17	80.0% (4)
**Ethnicity**				
Asian	25.0% (2)		Asian	40.0% (2)
White	37.5% (3)		White	40.0% (2)
Caucasian	37.5% (3)		Mixed	20.0% (1)

### Nominal group ranking

The PROMs prioritized by patients and family/caregivers before the discussion are displayed in Tables 2 and 3, respectively. We calculated the median and Interquartile range (IQR) for each PROM. Based on the median score and IQR, we ranked PROMs from top priority (highest median) to lowest priority (lowest median).

**Table 2:** Summary of NGT prioritization, parents/caregivers (N) = 8

Measures	P1	P2	P3	P4	P5	P6	P7	P8	Prioritization	
									Rank	Median (IQR)
Revised Child Anxiety and Depression Scale (RCAD-25)	8	10	8	10	9	10	10	3	1	9.5 (10–8)
Screen for Child Anxiety Related Disorders (SCARED)	8	10	8	7	9	10	3	7	2	8 (9.5–7)
The Young Person’s Core (YP-CORE)	6	7	7	8	10	8	10	7	3	7.5 (9–7)
Child Health Questionnaire (CHQ)	8	6	9	9	6	7	8	5	4	7.25 (8.5–6)
Pediatric Quality of Life Inventory (PedsQl)	7	8	10	2	2	5	10	7	5	7 (9–3.5)
Strength and Difficulties Questionnaire (SDQ)	6	7	8	4	5	10	8	7	6	7 (8–5.5)
Child Behaviour Checklist (CBCL)	7	7	8	3	6	10	6	7	7	7 (7.5–6)
Spence Children’s Anxiety Scale (SCAS)	8	5	7	6	5	10	8	4	8	6.5 (8–5)
Beck Depression Inventory (BDI)	8	5	8	0	8	10	3	4	9	6.5 (8–3.5)
KIDSCREEN-10	6	7	8	5	1	3	10	5	10	5.5 (7.5–4)

**Table 3:** Summary of NGT prioritization, patients (N = 5)

Measures	P1	P2	P3	P4	P5	Prioritization	
						Rank	Median (IQR)
Revised Child Anxiety and Depression Scale (RCAD-25)	10	7	10	6	9	1	9 (10–6.5)
Beck Depression Inventory (BDI)	10	6	9	10	6	2	9 (10–6)
The Young Person’s Core (YP-CORE)	10	6	9	8	7	3	8 (9.5–6.5)
Child Behaviour Checklist (CBCL)	10	4	8	9	7	4	8 (9.5–5.5)
Screen for Child Anxiety Related Disorders (SCARED)	10	8	8	3	6	5	8 (9–4.5)
Pediatric Quality of Life Inventory (PedsQl)	9	8	7	4	8	6	8 (8.5–5.5)
Spence Children’s Anxiety Scale (SCAS)	10	6	9	6	7	7	7 (9.5–6)
Strength and Difficulties Questionnaire (SDQ)	10	5	7	5	8	8	7 (9–5)
KIDSCREEN-10	9	9	4	1	7	9	7 (9–2.5)
Child Health Questionnaire (CHQ)	9	8	4	6	5	10	6 (8.5–4.5)

The highest prioritized PROMs among family/caregivers are the Revised Child Anxiety and Depression Scale (RCAD 25), with the highest median score (9.5) for being comprehensive, short, and brief, followed by Screen for Child Anxiety Related Disorders (SCARED). The Child Health Questionnaire (CHQ) is moderate on the choice among Family/caregivers, with a median score (7.25). Similarly, the KIDSCREEN become the least choice among family/caregivers because of generic measures.

While the RCAD 25 was aligned with the top prioritized PROs by family/caregivers, as the top prioritized PROM among patients with the highest median rank score (9) as being relevant, comprehensive, and brief, the Beck Depression Inventory (BDI) was at the second choice with the median rank score (9) rather than SCARED. Family/caregivers poorly ranked the BDI.

Both groups of participants ranked The Young Person’s Core (YP-CORE) in third place. In contrast, while the Child Health Questionnaire was ranked fourth by family/caregivers, it was ranked lowest by youth patients. The Spence Children’s Anxiety Scale, the longest and, therefore, most time-consuming to fill out, ranked poorly by both patients and family/caregivers (7).

### Re-ranking

After discussion, participants were asked if they wanted to revise their original ranking.

#### Family/caregiver priorities

After the discussion, the participants (family/caregivers) settled on the RCAD 25 as the best choice because of its relevancy, short form, and comprehensibility. The ranking order for the other two PROMs, SCARED and YP-CORE, remained the same.

A notable change in ranking was for Kidscreen 10 and CHQ, in which Kidscreen 10 increased in priority, and CHQ fell to the lowest rank after the discussion round as participants felt Kidscreen was short with ten questions and had good domain coverage. Some participants revealed that they liked the additional details asked in the SCAS. So, the participants decided to move SCAS higher in the ranking. Participants also discussed the importance of the aggression and rule-breaking behavior-related questions addressed in CBCL. This resulted in a change in the order of these PROMs. Participants also revealed a high preference for the PROMs that are rated by both parents and youths. Participants expressed that youth, especially teens, are ashamed of their feelings and some stigma around honesty in completing the questionnaire. Others stated that outcome measures should be filled out entirely by the patient independently, while some participants supported the idea of parents and youth filling out PROMs.

Further discussions revealed that the “negative” items in the questionnaires, relating to questions about the challenges and difficulties with mental health concerns, could be distressing. Two participants praised the Strength and Difficulties questionnaire for its content about positive mental health characteristics. The details regarding PROMs prioritization during NGT are summarized in the Table 4, and supporting quotations have been included in the Appendix.

**Table 4:** Summary of PROMs prioritized by family/caregivers and patients

Measures	Family/caregivers		Patients	
	Prioritization	Re-ranking	Prioritization	Re-ranking
RCAD 25	1	1	1	1
SCARED	2	2	2	5
YP-CORE	3	3	3	3
Kidscreen 10	10	4	9	9
SCAS	8	5	7	4
CBCL	7	6	4	6
PedsQl	5	7	6	7
SDQ	6	8	8	8
BDI	9	9	2	2
CHQ	4	10	10	10

#### Patients’ priorities

There were a few changes in the ranking of PROMs order after discussion. The rank order of top 1, 2, and 3 among the youth participants remained the same. After the discussion, participants reached an agreement with CBCL (rank 4, pre-discussion) and SCAS (rank 6, pre-discussion). They noted that CBCL was too long and could lose accuracy at the end; participants felt that SCAS needed to be ranked higher because it was more relevant and shorter. Most of the participants agreed to lower their prioritization of PedsQl as they assumed the items were non-relevant to them as it was viewed to be targeted to a younger population. The PROMS ranked 5, 8, 9, and 10 were consistent.

Views among the youth participants regarding the number of questions in the tools were highly variable. Most youth reported getting bored doing those questionnaires if there were 100 questions. However, some participants prefer longer questionnaires with easier answers that provide more in-depth information while in crisis. One youth participant stated that filling out the questionnaire is not just tedious but does not accurately reflect patient perspectives, as patients tend to answer negatively just because they feel annoyed by the questionnaire. Participants indicated they like to have a chat with clinicians/counselors instead.

**Fig. 3:**
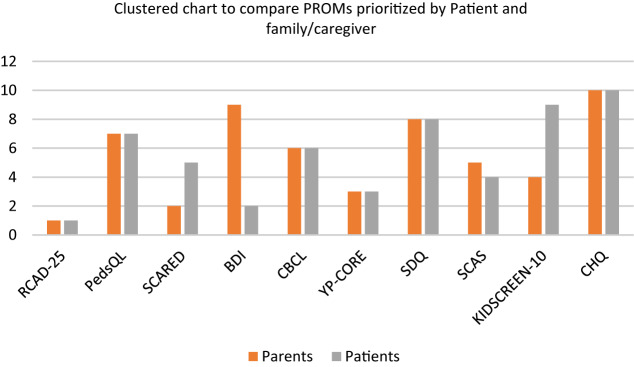
PROMs prioritized by family and patients. *Note* 1 = Highest priority, 10 = lowest priority

As shown in Fig. 3, the final rankings by patients and family/caregivers were the same for RCAD 25 (ranked as #1), YP-CORE (ranked #3), CBCL (ranked as #6), PedsQoL (ranked as #7), SDQ (ranked as #8), CHQ (ranked as #10). Both patients and family/caregivers highly prioritized RCAD 25 regarding feasibility, reliability, and overall importance. After discussion, NGT participants from both patients and family members felt that RCAD 25 was the best PROM because of its comprehensiveness (comprised both anxiety and depression, as well as obsessive-compulsive disorder) and because it was short and quick to complete.

## Discussion

We used a modified NGT to identify the relative priorities of pre-identified PROMs from the perspective of youth living with anxiety and/or depression and family/caregivers [20]. This study offered a novel and essential insight into integrating PROMs into clinical care to support youth mental health services. In general, the prioritized PROMs were broadly similar, with some minor differences in prioritizing priorities between patients and family/caregivers.

For patients and families/caregivers, the PROMs RCAD 25 and YP-CORE were the highest priorities. These priorities for PROMs reflected concerns about their relevance and feasibility. Both felt that RCAD 25 was comprehensive, short, easy, and quick to complete, whereas regarding YP-CORE, patients and family/caregivers thought it was also short and relevant. On the other hand, SDQ and CHQ were the lowest prioritized by patients and family/caregivers. The prioritization was underpinned by some reasons. Both thought the PROMs were too general, and most of the items needed to be more relevant to them.

There were some differences in the prioritization of PROMs by patients and family/caregivers. Based upon the relevance, family/caregivers gave high priority to SCARED, whereas it was lower prioritized among patients because patients thought it had too many items. Comparably, BDI was highly prioritized among the patients because they felt it was to the point and by length; it was neither too short nor too long. Surprisingly, parents/caregivers prioritized Kidscreen 10 higher because it is short and includes the items for assessing the quality of life. However, patients thought it was too general and irrelevant to them.

To our knowledge, this is one of the first studies to explore patients’ and family/caregiver perspectives on the relative priority of PROMs to be implemented in clinical care. Concerning the priorities, the ranking of PROMs did not alter markedly between the rounds in NGT among both groups. However, the discussion round led to greater consensus because the prioritized PROMs were deemed top priorities beyond the participants instead of having specific PROMs that were more important to the particular participants. Hence, it notifies that to comprehend the different perspectives and consensus building, the discussion was essential [32].

The study participants were quite clear that patient-reported measures are crucial to evaluate the outcome of treatments and interventions. They highlight the importance of parents/proxy-reported and self-reported questionnaires due to the stigma around youth not expressing their feelings so that parents would have more authentic feedback on behalf of their kids. Participants were concerned about the behavior and acting out related questions that do not necessarily indicate the patients’ struggle. This finding is consistent with the finding from Dowrick and colleagues, who found that some patients living with depression/anxiety may not answer the PROMs accurately to lessen stigma and/or influence treatment [33].

Our systematic approach led to the selection of disease-specific PROMs over generic PROMs. When we presented generic and disease-specific measures to NGT participants for prioritization, the benefit of disease-specific PROMs outweighed the generic PROMs; for instance, RCAD 25 was a priority, whereas CHQ was the least priority among the participants. This contradicts the previous study, which showed that NGT consensus for generic PROMs is opposed to arthritis-specific PROMs [34].

We found that participants were concerned with the overwhelming presence of negative items, which they felt would lead to inaccurate and invalid responses. This finding, accord to Marsh, presumed that younger children respond in polar extremes to negatively worded items because they require more verbal reasoning than positively worded items [35]. One solution to this might be the use of balanced scales in PROMs. That is, ensuring that scales have an equal number of positive and negative items [36].

In our study, participants noted concerns about the lengths of various questionnaires. Others have also indicated similar circumstances surrounding the acceptability of questionnaires based on length [25, 37]. A study by Kost et al. showed that shorter questionnaires are reliable and have higher responses and completion rates than lengthier questionnaires [38]. As the number of questions increases, participants tend to rush or skip over some of the items to get over it, adversely impacting the accuracy, reliability, and response rate [39].

The evidence suggested that the generic measures may not be suitable for those living with mental health concerns; there is also less evidence to support their appropriateness in this population, such as anxiety, depression, and so on [40, 41]. While the outcome measure such as PedsQl has been precisely developed to assess the health-related quality of life, the youth participants from our study conveyed the view that this measure was inadequately detailed to portray the quality of life of youth living with mental illness. Conversely, the family/caregivers articulated that they prefer Kidscreen 10, which is short and includes the items for assessing the quality of life. Remarkably, some participants also raised concerns about gender-based questionnaires, as identical questionnaires could not be effective for males, females, and non-binary because they are all present in very different ways.

This study is not without limitations. Both patients and family/caregivers were recruited via one research registry, which might affect the generalizability of the results to other clinical settings. In our NGT session, only one male participant in the family/caregiver group, and none of the participants in the youth patient group were male. Due to the small sample size, our findings may not represent the views of all youth patients living with anxiety and/or depression and family/caregivers [20]. However, the sample size is inherent to the NGT, a small group consensus-building method. We adhered to NGT standards, including five to eight participants [20, 42]. Having more than 10 participants per group may inhibit the open discussions needed in NGT, owing to the size of the group [43]. Lastly, although we included the measures that have been widely validated and used in youth mental health, focusing on anxiety and depression, others may have been missed in our pre-identified list of PROMS.

Study strengths include the use of qualitative and quantitative approaches to elicit patients’ and family members’ priorities and understand the reason for their prioritization. Qualitative data, audio transcribed verbatim and thematically analyzed, provided richness around PROMs’ preferences, relevance, acceptability, and overall feasibility. Furthermore, we employed a pragmatic approach to sort out the PROMs, where we could attain diverse representation in mapping and selecting the PROMs, representing clinicians, administrators, family advisory groups, and youth partners from Alberta. Finally, we conducted separate NGTs for patients and family/caregivers to ensure both perspectives were captured and taken into account.

## Conclusion

The importance of incorporating patients’ and family/caregivers’ perspectives has been recognized in healthcare settings and research. This study provides practical recommendations around measures best suited to achieve this. The healthcare professional can incorporate these PROMs in their routine patient assessment and management. Additionally, these PROMs provide valuable information that might be utilized to address the concerns about the treatment efficacy, help to evaluate disease progression and regression, as well as the value of care provided [44], and help to identify the patients who may be at risk and allow the healthcare professional to evaluate the quality of their care continuously [45]. Healthcare professionals and policymakers can be guided by the patient-family/caregivers’ voices captured through consensus methodologies such as NGT to implement the PROMs relevant to patients and family members in mental health settings.

## Data Availability

All data generated and analyzed during this review are included in this published article.

## Appendix

**Table 5:** Summary of PROMs prioritized by family/caregivers and patients

Measures	Family/caregivers		Patients		Quotes
	Prioritization	Re-ranking	Prioritization	Re-ranking	
PedsQl	5	7	6	7	“I think there is more important values to rank higher than pediatric then that one at that point at this point as its too general”. (Family/Caregiver) “I would lower down the pediatrics one because me personally when I went through that question it kind of felt a bit surprising I just I didn’t find myself like relating to many of the questions related to elementary”. (Patients)
Kidscreen 10	10	4	9	9	“It’s a short, quick 10 questions, and the physical activity and energy are good questions. There’s a lot of good ones regarding, relationships with parents and peers, school functioning”. (Family/Caregiver)
SDQ	6	8	8	8	(Family/caregiver felt SDQ should swap with CBCL)
CHQ	4	10	10	10	“I agreed with that CHQ one just lowered down just because it was so general and broad that I and a lot of questions, too I think, I felt like it was. It should be lower on the on the scale”. (Family/Caregiver)
CBCL	7	6	4	6	“It covers a lot and the aggressive and rule breaking behaviour is different than the other. Lots of questions so, obviously there’s a lot of little things that you need to click or mark off, so thought that it covered a broad spectrum of items that you would need to look at for a child’s behavior. It should be replaced with Strength and difficulty one” (Family/Caregiver)
					“I get bored doing those. I put. I think the child behavior check was lower because it was like 100 questions like I could not because if it’s too long, then you’ll just start like answering the questions to get it over with”. (Patients)
SCARED	2	2	5	5	No discussion
SCAS	8	5	7	4	“I am quite surprised about SCAS being ranked so lower. It is more specific, covers Obsessive compulsive and covers a physical injury, fear which is very important to consider as the self harm is on the increase especially girls are self harming”. (Family/Caregiver)
BDI	9	9	2	2	“I choose BDI number two because they found like the questions like, I guess it’d be like a medium like size, like kind of question, and to the point and its relevant to me as I suffer a lot with depression”. (Patients)
RCAD 25	1	1	1	1	‘I rated Number One was that it was RCAD, because it’s a fairly short and quick measure. I think it covers a lot of questions, I guess they cover a lot of about Anxiety as well as depression in one measure.’ (Family/Caregiver)
					‘I struggle with anxiety and depression most of my childhood, so RCAD measures both in one questiionaire. So, it’s a short, quick and relevant measure’. (Patients)
YP-CORE	3	3	3	3	“I think, like the YP Core like question. There it is the 10 like questions. One, right? I find it like to be like pretty effective in like getting like the right points in a like quick a way. So, like since people like my feel differently about like filling in questions, I still think the YP core is like an important one. A lot of they go over to us”. (Patients)

## Abbreviations

MHR 4 Kids: Mental Health Research for Kids Research registry
MBC: Measurement-based care
RCAD-25: Revised Child Anxiety and Depression Scale
SCARED: Screen for Child Anxiety Related Disorders
YP-CORE: The Young Person’s Core
CHQ: Child Health Questionnaire
PedsQl: Pediatric Quality of Life Inventory
SDQ: Strength and Difficulties Questionnaire
CBCL: Child Behaviour Checklist
SCAS: Spence Children’s Anxiety Scale
BDI: Beck Depression Inventory
